# From Screening to Therapy: A Personalized Approach to ROP in a National NICU Setting

**DOI:** 10.3390/jpm15080388

**Published:** 2025-08-19

**Authors:** Stylianos Christodoulou, Fedonas Herodotou, Annalisa Quattrocchi, Theodoros Potamitis, Vivi Choleva

**Affiliations:** 1 Ophthalmology Department Makarios Hospital, Nicosia 2011, Cyprus; vivi.choleva@gmail.com; 2 Medical School, University of Cyprus, Nicosia 2029, Cyprus; fedonascom@gmail.com; 3 Department of Primary Care and Population Health, University of Nicosia Medical School, Nicosia 2408, Cyprus; quattrocchi.a@unic.ac.cy; 4 Pantheo Eye Centre, Limassol 3076, Cyprus; theodoros@cytanet.com.cy

**Keywords:** type 1 ROP, Cyprus, anti-VEGF, laser photocoagulation, prematurity, neonatal risk factors, ROP screening

## Abstract

**Aim**: We aimed to investigate the incidence, treatment patterns, and associated risk factors of type 1 retinopathy of prematurity (ROP) in the only tertiary-level Neonatal Intensive Care Unit (NICU) in Cyprus. **Methods**: This retrospective study included all infants screened for ROP between January and December 2023. Data were collected from standardized NICU discharge summaries and included gestational age (GA), birth weight (BW), multiple birth, systemic infection, blood transfusion, oxygen therapy, surgical interventions, and ROP outcomes. Infants were categorized into non-ROP, non-type 1 ROP, and type 1 ROP groups. Statistical analysis was performed to identify differences in risk factor distribution. **Results**: Among 183 infants, 33 (18.0%) developed ROP, with 11 (6.0%) requiring treatment for type 1 ROP. All infants with type 1 ROP were born at ≤28 weeks GA and weighed <1501 g. Type 1 ROP was significantly associated with lower GA, lower BW, systemic infection, surgery, and prolonged oxygen support (*p* < 0.05). Six infants were treated with laser and three with intravitreal bevacizumab. No recurrence was observed in the anti-VEGF group during 18 months of follow-up. Two infants with aggressive ROP died before treatment. **Conclusions**: Type 1 ROP in Cyprus occurred exclusively in extremely preterm infants, associated with the cumulative effect of multiple risk factors. Laser remained the primary treatment, while anti-VEGF was used selectively with favorable outcomes. This study emphasizes the importance of tailoring ROP screening and treatment strategies based on individual neonatal risk profiles, supporting a personalized approach to neonatal ophthalmic care.

## 1. Introduction

Retinopathy of prematurity (ROP) is a worldwide leading cause of childhood blindness, resulting from disrupted retinal vascular development in preterm infants. As neonatal care advances globally, the survival of extremely premature infants has improved, yet this has also contributed to a rising incidence of severe forms of ROP. The pathophysiology of ROP involves two distinct phases. In the first phase, hyperoxia due to preterm birth suppresses vascular endothelial growth factor (VEGF), halting retinal vessel growth. The subsequent hypoxic phase leads to VEGF overexpression, triggering abnormal fibrovascular proliferation and potentially progressing to tractional retinal detachment if untreated [1,2].

Type 1 ROP represents the threshold at which treatment is clinically indicated and is defined by the Early Treatment for ROP (ETROP) criteria as stage 3 ROP in zone I or II with plus disease, or any stage ROP in zone I with plus disease. This classification reflects an aggressive disease course with a high risk of progression, requiring prompt intervention. Management strategies for type 1 ROP have evolved significantly in recent decades. Laser photocoagulation, once the mainstay of treatment, ablates avascular peripheral retina to reduce VEGF production and stabilize neovascularization. However, this technique compromises peripheral visual fields and increases the risk of high myopia [3].

In recent years, anti-VEGF agents like bevacizumab, ranibizumab, and aflibercept have emerged as alternatives or adjuncts to laser therapy. These agents target VEGF more selectively, allowing for continued peripheral retinal vascularization and resulting in less refractive error. Studies demonstrate their efficacy, particularly for aggressive ROP (A-ROP), though concerns remain about potential systemic effects due to VEGF’s role in organogenesis and neurodevelopment [2,4,5].

Despite these advancements, the management of type 1 ROP remains complex, influenced by disease severity, anatomical location, and patient-specific factors. Both modalities have demonstrated efficacy, but they carry unique risks and benefits. Laser therapy is associated with greater structural stability in the long term, whereas anti-VEGF agents require careful follow-up due to the risk of late reactivation [6,7].

In Cyprus, neonatal care is centralized in a single tertiary Neonatal Intensive Care Unit (NICU), providing a unique opportunity to examine national ROP trends. To date, no comprehensive epidemiological data exist on the incidence, risk factors, and treatment outcomes of type 1 ROP in this setting. This study addresses this gap by investigating the characteristics and therapeutic patterns of ROP in Cyprus, with a particular emphasis on infants who required treatment for type 1 disease. Our findings aim to inform future clinical guidelines and support the development of personalized screening strategies in similar healthcare environments.

There is a growing recognition that neonatal care, particularly in managing ROP, benefits significantly from personalized medicine. Individual variability in gestational maturity and systemic comorbidities necessitate a tailored approach to screening frequency and therapeutic interventions. This study contributes to the understanding of how personalized neonatal care can optimize outcomes in a real-world NICU setting.

## 2. Methods

### 2.1. Study Design and Data Collection

This study was a single-center, retrospective cohort study conducted at the NICU in Makarios Hospital. This study included all infants who underwent ROP screening between 1 January and 31 December 2023.

This centralized setting allowed us to capture a comprehensive national cohort of preterm infants at risk of ROP, minimizing selection bias and ensuring population-level relevance.

### 2.2. ROP Screening Criteria

ROP screening was conducted according to institutional guidelines based on the UK Royal College of Ophthalmologists and Royal College of Paediatrics and Child Health (2008) recommendations [8]. Although updated UK guidelines were released in 2022, our institution’s screening protocol during the study period was still based on the 2008 recommendations, which were in active use throughout 2023. The inclusion criteria for screening were

- Gestational age (GA) at birth <32 weeks;
- Birth weight (BW) < 1501 g.

Additionally, neonatologists could refer infants with GA ≥ 32 weeks or BW ≥ 1501 g for screening based on clinical concerns.

All examinations were performed by a single experienced pediatric ophthalmologist (VC), ensuring consistency in the diagnostic classification and reducing inter-observer variability.

### 2.3. Data Collection

Data were extracted from standardized NICU discharge summaries and electronic medical records. These documents provided structured information on demographics, perinatal risk factors, systemic complications, and ROP screening outcomes.

Collected variables included the following:

- Gestational age (in weeks) and birth weight (in grams);
- Type of pregnancy (singleton vs. multiple birth);
- Ethnic background;
- Systemic infections (defined as laboratory-confirmed sepsis);
- Blood transfusions;
- Surgeries (under general anesthesia);
- Duration of oxygen therapy (total hours on supplemental oxygen via mechanical ventilation, CPAP, or nasal cannula);
- ROP outcomes, including zone, stage, presence of plus disease, and treatment modality.

To ensure consistency, two independent investigators extracted data, and discrepancies were resolved through consensus with a third reviewer.

### 2.4. Classification of ROP and Treatment Protocol

The ROP classification followed the International Classification of ROP (ICROP) and UK screening and treatment guidelines updated in 2022 [9]. Infants were divided into three categories based on the most severe form of ROP observed during the screening period:

- Non-ROP;
- Non-type 1 ROP (defined as the presence of any type of ROP not requiring treatment);
- Type 1 ROP (defined as per ETROP criteria: zone I any stage with plus, zone I stage 3, or zone II stage 2 or 3 with plus disease).

Treatment decisions were made based on the anatomical location and severity of disease:

Laser photocoagulation was the first-line treatment for zone II stage 2 or 3 with plus disease. Treatment was performed using transpupillary green 532 nm laser photocoagulation (Visulas) in a near-confluent pattern.

Intravitreal anti-VEGF injection (bevacizumab 0.625 mg/0.025 mL) was used selectively for aggressive ROP (A-ROP) in cases involving zone I.

No other anti-VEGF agents were available in Cyprus during the study period.

Follow-up after treatment was individualized, with increased frequency during the first 3 months and extended follow-up beyond 18 months for anti-VEGF-treated infants, consistent with UK and international recommendations.

### 2.5. Statistical Analysis

Numerical variables were expressed as means ± standard deviation (SD) and categorical variables as percentages. Categorical variables were created for gestational age at birth (GA): group with GA ≤ 31 weeks + 6 days and group with GA > 32 weeks.

The study participants were divided into three subgroups based on the outcome: infants diagnosed with non-ROP, non-type 1 ROP, and type 1 ROP.

The incidence and the course of ROP in different GA categories were described by descriptive statistics.

Chi-square or Fisher’s exact test for categorical variables and one-way ANOVA for continuous variables were used to compare differences between the groups.

All analyses were conducted using Stata version 18 (StataCorp, College Station, TX 77845, USA). Statistical significance was defined as a *p*-value < 0.05.

### 2.6. Ethical Approval

The current study has been registered and was approved by the National Bioethics Committee in Cyprus (n. 2023.01.143), and it was conducted according to the principles of the Declaration of Helsinki. The data were collected anonymously.

## 3. Results

In total, 183 patients were included in this study with a mean gestational age (GA) of 30.2 ± 2.7 weeks and a mean birth weight (BW) of 1382.5 ± 423.0 g. The incidence of ROP was 18.0% (N = 33). At a mean postmenstrual age of 34.3 ± 2.3 weeks, 22 infants developed non-type 1 ROP (66.6%) and 11 (33.3%) developed type 1 ROP and required treatment bilaterally. Sadly, two infants with type 1 ROP passed away before receiving treatment.

In the three subcategories, the mean GA in weeks was 31.0 ± 2.1 for non-ROP, 27.2 ± 2.4 for non-type 1 ROP, and 25.4 ± 1.5 for type 1 ROP. Additionally, the mean BW in grams was 1493.5 ± 353.8 for non-ROP, 966.4 ± 372.9 for non-type 1 ROP, and 701.6 ± 47.0 for type 1 ROP (Table 1).

There was a statistical difference between the three groups for BW < 1501 g, GA ≤ 31 weeks + 6 days, infections, surgery, and prolonged use of oxygen (*p* < 0.05).

**Figure 1:**
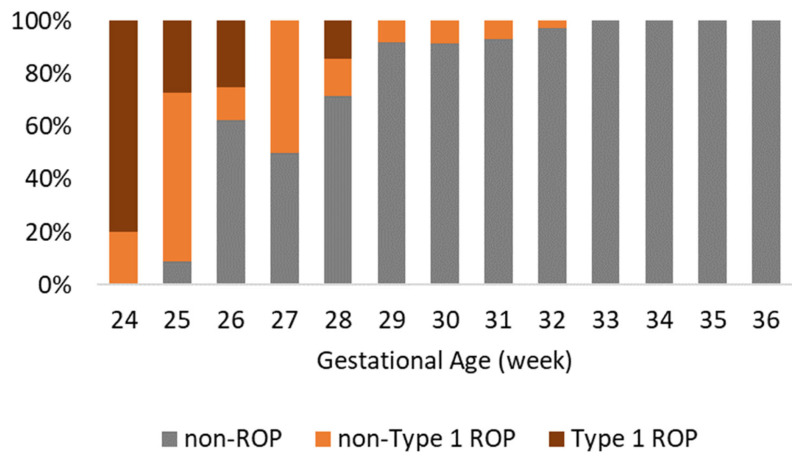
ROP severity trends across gestational ages at birth.

As Figure 1 illustrates, the incidence and severity of ROP decrease sharply with increasing gestational age, with treatment-requiring ROP (type 1 ROP) confined almost entirely to infants born at ≤28 weeks, with the highest proportion at 24 weeks, where it accounts for nearly 80.0% of cases.

Among the eleven infants diagnosed with type 1 ROP, six received laser photocoagulation, for stage 3 with plus disease in zone 2, three were treated with intravitreal anti-VEGF injections for aggressive ROP, and two with aggressive ROP unfortunately passed away prior to receiving treatment.

For the laser-treated group, the mean postmenstrual age at ROP onset was 34.33 ± 1.03 weeks (range: 33–36 weeks), and the mean age at treatment was 41.00 ± 3.22 weeks (range: 37–46 weeks). Five cases had only one course of laser treatment, and one had additional laser treatment within 2 weeks from the first treatment.

The three infants who received anti-VEGF injections had a mean ROP onset at 34.00 ± 1.00 weeks (range: 33–35 weeks), and treatment was administered at a mean of 36.00 ± 0.00 weeks. The two deceased infants were diagnosed with aggressive posterior ROP at 34 and 38 weeks postmenstrual age, respectively. All three infants who received intravitreal bevacizumab injections showed no recurrence of ROP over the subsequent 18 months. Retinal vascularization was completed after several months, with a mean time of 8.17 ± 0.76 months.

**Figure 2:**
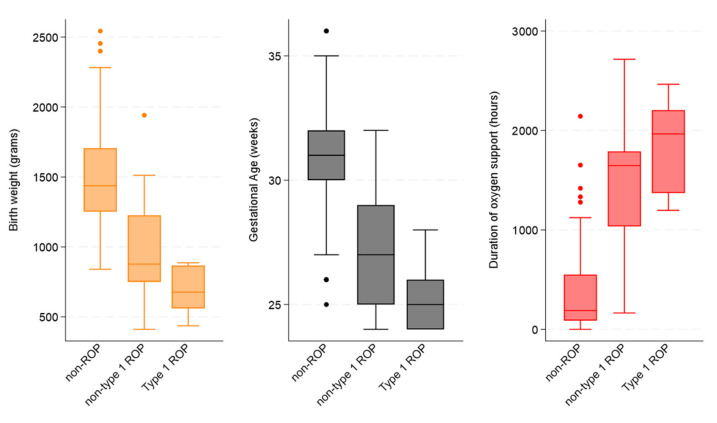
Distribution and comparison of birth weight (orange), gestational age (grey), and duration of oxygen support (red) by ROP category.

Furthermore, the statistical difference amongst the three groups for BW, GA, and duration of oxygen support can be seen in Figure 2 (all *p*-values < 0.001).

However, when non-type 1 ROP cases were compared with type 1 ROP cases, the only statistical difference was found for mean BW (966.4 ± 372.9 vs. 701.6 ± 47.0; *p* = 0.032) and mean GA (27.2 ± 2.4 vs. 25.4 ± 1.5; *p* = 0.024).

It is also noteworthy that, in our cohort, 12 infants had severe intrauterine growth retardation and low postnatal weight gain. Among them, three developed type 1 ROP and required treatment, while another four developed ROP but did not require treatment.

## 4. Discussion

This study offers valuable epidemiological data on type 1 ROP in Cyprus, focusing on the prevalence, treatment outcomes, and associated risk factors in the country’s only tertiary NICU. Among 183 screened babies, the incidence of any ROP was 18.0% and of type 1 ROP was 6.0%. These figures align closely with those from developed nations such as the USA, Spain, and the UK. For instance, the G-ROP study reported a 12.4% treatment rate for type 1 ROP, while Spanish and UK studies documented treatment incidences of 14.6% and 13.8%, respectively [10,11,12].

Large-scale analyses confirm that almost all treatment-requiring ROP cases occur in the smallest and most premature infants. In the G-ROP cohort, only 0.7% of severe ROP cases were in infants with a birth weight >1251 g, supporting the selective but vigilant screening of high-risk neonates [12]. Our finding that all type 1 ROP cases occurred in ≤28-week infants mirrors other national datasets, where gestational age and birth weight remain the strongest predictors of severe disease [10].

In our study, two treatment modalities were utilized: laser photocoagulation and intravitreal bevacizumab. Laser photocoagulation, the standard treatment for stage 3 ROP in zones 2 and 3, was the predominant choice. Laser is a well-established treatment with a low complication rate and proven safety profile [13,14].

Intravitreal bevacizumab was reserved for aggressive ROP (A-ROP), a rapidly progressing condition. Bevacizumab-treated infants in our cohort demonstrated complete retinal vascularization without recurrence in a 18-month follow-up, similar to findings from Spain, where anti-VEGF therapy showed low recurrence rates and favorable outcomes [1,10,15]. In this one-year review, there was ratio of one case of intravitreal injection to two laser treatments.

Due to the risk of late ROP reactivation and serious visual consequences, extended and diligent follow-up is essential after anti-VEGF therapy. Many experts recommend monitoring infants beyond traditional screening timelines (frequent follow-up to 18 months after treatment and then 6-monthly or annually up to the age of 5 years according to UK ROP treatment guidelines), ensuring that reactivation is caught early before irreversible damage occurs. It is a quick procedure, and selected cases respond well with a high safety profile compared to laser. However, it significantly increases the follow-up workload. As infants grow older, it also becomes increasingly difficult to examine the far retinal periphery [13,14].

Recent studies provide interesting insights into treatment approaches. For instance, Sen et al. (2021) introduced the outcomes of combining anti-VEGF injection and laser photocoagulation in type 1 ROP, concluding that this strategy was effective and safe, potentially reducing the need for the strict long-term follow-up often required with monotherapy [16]. In parallel, Tung et al. (2024) provide additional longitudinal insights, reporting that while ranibizumab-treated eyes showed a myopic shift and longer axial lengths at age three compared with bevacizumab, these differences diminished by age six due to emmetropization [17]. This indicates that early biometric disparities may not translate into long-term refractive disadvantages, though vigilant refractive monitoring remains warranted [17]. Furthermore, a meta-analysis of neurodevelopmental outcomes reported no statistically significant increase in severe neurodevelopmental impairment after intravitreal bevacizumab compared with laser or untreated controls; however, it did reveal a small reduction in Bayley-III motor scores, underscoring the importance of long-term multidisciplinary follow-up [18]. Given the ongoing discussion regarding the systemic effects of anti-VEGF agents like bevacizumab, the inclusion of long-term neurodevelopmental follow-up would be valuable in future studies to assess potential implications on overall infant development.

In our study, risk factor analysis identified low gestational age, low birth weight, and prolonged oxygen therapy as significant contributors to ROP development and progression. These findings are consistent with international studies, which emphasize gestational age and birth weight as dominant risk factors. The G-ROP study reported that infants with gestational ages ≤24 weeks or birth weights ≤500 g had the highest odds of developing type 1 ROP [12], similarly to our cohort, of which the group comprising 24 weeks of gestational age infants has the highest risk of ROP type 1. Consistent with our results, Gaber et al. (2021) identified low gestational age, low birth weight, and prolonged oxygen supplementation as independent predictors of type 1 ROP [19]. Interestingly, this study found an overall incidence of ROP of 34.1%, with type 1 ROP occurring in 26.3% of infants, and additionally suggested apnea and thrombocytopenia as significant associated factors. These results reinforce the multifactorial nature of disease pathogenesis, where both hypoxic–ischemic and inflammatory pathways are implicated [19]. Importantly, moving beyond these well-established clinical parameters, recent attention has shifted towards novel biomarkers. In particular, thrombocytopenia at birth (<181 × 10^9^/L) and a characteristic pre-treatment platelet “spike” have been linked to an increased risk of type 1 ROP. Incorporating such laboratory markers into predictive algorithms could substantially enhance screening specificity and support the personalized scheduling of follow-up examinations [20].

Our findings further suggest that the cumulative presence of multiple risk factors exacerbates disease severity, particularly in cases of type 1 ROP. The Spanish study identified late-onset sepsis, mechanical ventilation, and weight at 28 days as independent predictors of ROP progression [10]. Other additional risk factors, for which their role needs to be elucidated further, are maternal pre-eclampsia and gestational diabetes [21], maternal age and emotional stress [22], premature rupture of membranes and chorioamnionitis, higher level of oxygen and fluctuation of oxygen levels, necrotizing enterocolitis, intraventricular hemorrhage, and poor postnatal weight. Some of these results seem to be contradictory in clinical studies due either to different and heterogenous preterm infant populations or different neonatal care across the world. In addition to biological and neonatal factors, broader determinants such as maternal health and socioeconomic status—although not included in our analysis—may also influence ROP development. These aspects, typically managed within neonatal care systems, warrant further investigation in future interdisciplinary research. This study has some limitations. Its retrospective design and relatively small sample size, especially for treated cases, limit the generalizability of the findings. The lack of long-term follow-up data, particularly for infants treated with anti-VEGF therapy, precludes definitive conclusions about treatment safety and durability. Nonetheless, this study establishes a strong foundation for understanding type 1 ROP trends in Cyprus and highlights areas for future research and clinical improvements. Moreover, future studies conducted under the updated 2022 UK guidelines may yield different screening outcomes, particularly with regard to inclusion criteria and follow-up recommendations.

## 5. Conclusions

This one-year review of type 1 retinopathy of prematurity in Cyprus provides the first national insight into the epidemiology, treatment patterns, and risk factors associated with type 1 ROP in a centralized tertiary NICU setting.

This study confirms that lower gestational age and birth weight are the strongest predictors of ROP severity, with type 1 ROP occurring exclusively in infants born at ≤28 weeks. Additional significant associations were found with systemic infections, prior surgeries, and the need for prolonged oxygen support. The findings also highlight the cumulative effect of multiple risk factors, reinforcing the importance of comprehensive neonatal care and early screening.

Laser photocoagulation remained the most frequently used treatment modality, while anti-VEGF was reserved for aggressive ROP cases. Both treatments were effective in halting disease progression, with no recurrence observed during an 18-month follow-up in the anti-VEGF group.

These findings support the ongoing need for tailored screening strategies, resource allocation, and long-term monitoring to optimize outcomes for preterm infants in Cyprus.

Our findings have several practical implications for neonatal ophthalmic care. Type 1 ROP developed exclusively in extremely preterm infants with additional systemic risk factors highlights the value of risk-adapted screening protocols beyond gestational age or birth weight alone. The favorable outcomes observed with both laser and anti-VEGF therapy support their continued use, with anti-VEGF reserved for selected cases where laser is less suitable. Given the extended follow-up required after anti-VEGF treatment, structured post-treatment monitoring is essential. The recent approval of aflibercept for ROP treatment in Cyprus’ General Health System as an alternative to bevacizumab introduces new opportunities for individualized treatment, outcome comparisons, and future clinical audits. Finally, the centralized care model in Cyprus offers a strong basis for developing a national ROP registry, which could support quality improvement and personalized screening strategies.

In conclusion, this study provides a comprehensive understanding of type 1 ROP in Cyprus, serving as a foundation for future research and local healthcare improvements.

This study underscores the relevance of personalized medicine in neonatal ophthalmology, advocating for risk-based screening and tailored treatment protocols to improve ROP outcomes in diverse populations.

## Figures and Tables

**Table 1:** Patients’ characteristics and risk factors’ distribution among non-ROP, non-type 1 ROP, and type 1 ROP.

Patients’ Characteristics	Non-ROP N = 150	Non-Type 1 ROP N = 22	Type 1 ROP N = 11	*p*-Trend *
	n (%)	n (%)	n (%)	
**Birth weight in grams (mean ± SD)**	1493.5 ± 353.8	966.4 ± 372.9	701.6 ± 47.0	**<0.001**
**Birth weight < 1501 g**				
No	64 (42.7)	2 (9.1)	0 (0)	**<0.001**
Yes	86 (57.3)	20 (90.9)	11 (100)	
**Gestational week (mean ± SD)**	31.0 ± 2.1	27.2 ± 2.4	25.4 ± 1.5	**<0.001**
**Gestational week ≤ 31 + 6 days**				
No	70 (46.7)	1 (4.5)	0 (0)	<0.001
Yes	80 (53.3)	21 (95.5)	11 (100)	
**Multiple births**				
No	94 (82.5)	15 (68.2)	6 (54.5)	0.743
Yes	56 (82.4)	7 (31.8)	5 (45.5)	
**Race**				
African	13 (8.7)	1 (4.5)	1 (9.1)	0.804
Asian	9 (6.0)	2 (9.1)	1 (9.1)	
Caucasian	128 (85.3)	19 (86.4)	9 (81.8)	
**Infection**				
No	96 (95.0)	4 (18.2)	1 (9.1)	**<0.001**
Yes	54 (65.9)	18 (81.8)	10 (90.9)	
**Blood transfusion**				
No	30 (96.8)	1 (4.5)	0 (0)	0.081
Yes	120 (79.0)	21 (95.5)	11 (100)	
**Surgery**				
No	141 (83.9)	19 (86.4)	8 (72.7)	**0.021**
Yes	9 (60.0)	3 (13.6)	3 (27.3)	
**Oxygen support**				
No	9 (100)	0 (0.0)	0 (0.0)	0.777
Yes	141 (81.0)	22 (94.0)	11 (100)	
**Oxygen duration in hours (mean ± SD)**	362.9 ± 380.3	1513.4 ± 743.9	1844.0 ± 1549.7	**<0.001**

* values in bold font indicate statistical significance (*p* < 0.05).

## Data Availability

The data supporting the findings of this study are available from the corresponding author upon reasonable request.
